# Non-lethal genotyping of *Tribolium castaneum* adults using genomic DNA extracted from wing tissue

**DOI:** 10.1371/journal.pone.0182564

**Published:** 2017-08-11

**Authors:** Frederic Strobl, J. Alexander Ross, Ernst H. K. Stelzer

**Affiliations:** Physical Biology / Physikalische Biologie (IZN, FB 15), Buchmann Institute for Molecular Life Sciences (BMLS), Cluster of Excellence Frankfurt–Macromolecular Complexes (CEF–MC), Goethe Universität–Frankfurt am Main (Campus Riedberg), Max-von-Laue-Straße 15, Frankfurt am Main–Germany; University of Thessaly School of Agricultural Sciences, GREECE

## Abstract

The red flour beetle *Tribolium castaneum* has become the second most important insect model organism and is frequently used in developmental biology, genetics and pest-associated research. Consequently, the methodological arsenal increases continuously, but many routinely applied techniques for *Drosophila melanogaster* and other insect species are still unavailable. For example, a protocol for non-lethal genotyping has not yet been adapted but is particularly useful when individuals with known genotypes are required for downstream experiments. In this study, we present a workflow for non-lethal genotyping of *T*. *castaneum* adults based on extracting genomic DNA from wing tissue. In detail, we describe a convenient procedure for wing dissection and a custom method for wing digestion that allows PCR-based genotyping of up to fifty adults in less than an afternoon with a success rate of about 86%. The amount of template is sufficient for up to ten reactions while viability and fertility of the beetles are preserved. We prove the applicability of our protocol by genotyping the *white / scarlet* gene pair alleles from the black-eyed San Bernadino wild-type and white-eyed Pearl recessive mutant strains spanning four generations. Non-lethal genotyping has the potential to improve and accelerate many workflows: Firstly, during the establishment process of homozygous cultures or during stock keeping of cultures that carry recessively lethal alleles, laborious test crossing is replaced by non-lethal genotyping. Secondly, in genome engineering assays, non-lethal genotyping allows the identification of appropriate founders before they are crossed against wild-types, narrowing the efforts down to only the relevant individuals. Thirdly, non-lethal genotyping simplifies experimental strategies, in which genotype and behavior should be correlated, since the genetic configuration of potential individuals can be determined before the actual behavior assays is performed.

## Introduction

During the past century, the fruit fly *Drosophila melanogaster* has become the best understood insect and an established model organism in developmental biology and genetics [1]. However, *D*. *melanogaster* is a highly specialized species and thus considered non-representative of insects in general. In contrast, the red flour beetle *Tribolium castaneum* (Herbst, 1797), an emerging insect model organism [2,3], is considered more generic since it retained many ancestral features [4,5]. During embryonic development, both species differ remarkably in their principles of body segmentation [6–8], organization of extra-embryonic membranes [9,10] and formation of appendages [11,12]. Since *T*. *castaneum* is of agricultural and thus also of economic importance [5,13], considerable research focuses on pest control measures [14,15].

For almost two decades, the methodological arsenal for *T*. *castaneum* has increased steadily [16]. Currently, it covers procedures such as non-invasive long-term live imaging with light sheet-based fluorescence microscopy [17–20], embryonic [21], larval [22] and parental [23,24] RNA interference, comprehensive *in situ* hybridization and antibody staining protocols [25,26], germline transformation with the lepidopteran *piggyBac* [27,28] or the dipteran *Minos* [29] transposon and genome engineering with the CRISPR/Cas9 system [30].

During certain experimental workflows, for example those involving mutant, transgenic or genetically engineered lines, it is usually necessary to determine the genotype of individual beetles. In certain scenarios, the genotype can be identified by (i) the phenotype, (ii) familiarity with the parental genotypes, (iii) test crossing, or (iv) a combination thereof. However, the phenotype typically allows only the partial identification of the genotype, the parental genotypes are not always known and test crossing requires manpower and time and may interfere with the overall experimental strategy. Alternatively, individual beetles can be sacrificed to extract their genomic DNA. This allows an evaluation of the genotype with molecular biology-based assays, but the respective individuals are unavailable for downstream experiments or crossing assays. In summary, both approaches suffer from limitations.

Non-lethal genotyping, which is standard for vertebrate model organisms such as mouse and zebrafish, determines the genotype while retaining the individual. PCR-based genotyping, one of the most commonly performed procedures, requires a small amount of blood or tissue, from which genomic DNA is extracted. Similar protocols for insect model organisms have been developed for *D*. *melanogaster* [31] and the honeybee *Apis mellifera* [32] by using dissected wing tissue as the DNA source. In both studies, the procedure is invasive, but affected individuals (females and males for *D*. *melanogaster*, queens for *A*. *mellifera*) have the same mating success as control animals.

Research on *T*. *castaneum* is strongly driven by genetic approaches: the genome has been sequenced [13] and massive data from large-scale projects such as insertional mutagenesis [33] and systematic RNA interference gene knockdown [34,35] screens are available. Non-lethal genotyping for *T*. *castaneum* has not yet been established but is particularly useful for the generation of homozygous lines, to facilitate stock keeping of lines that carry recessive lethal alleles, to identify appropriate founders during genome engineering assays and to preselect candidates with known genotypes in behavior assays.

In this study, we present a convenient workflow for non-lethal genotyping of *T*. *castaneum* adults by using wing tissue as the genomic DNA source and prove the applicability of our technique in more than one hundred individuals. We explain in detail how wings can be dissected without affecting survival and fertility and outline a custom method for genomic DNA extraction that performs reliably in PCR-based assays. The amount of DNA per wing is sufficient for up to ten reactions and single reactions yield enough PCR product for sequencing. Furthermore, we obtain the nucleotide sequence of the ABC transmembrane transporter *white* from the two prominent laboratory background strains San Bernadino (SB) and Pearl (Prl) and generate respective hybrids to perform a four generation proof-of-principle assay with phenotypic validation. Our method complements the arsenal of molecular biology techniques that are available for *T*. *castaneum* and can be used to simplify comprehensive workflows.

## Materials and methods

### *Tribolium castaneum* rearing and crossing

The *Tribolium castaneum* black-eyed SB wild-type [36] and the white-eyed Prl recessive mutant [37] strains were used in this study. Rearing was performed as described previously [5]. In brief, stock cultures were kept on full grain wheat flour (113061036, Demeter) supplemented with 5% (wt/wt) inactive dry yeast (62–106, Flystuff/Genesee Scientific) in 1-liter glass bottles at 32°C and 70% relative humidity in a 12-h light/12-h darkness cycle. Individuals were sexed as pupae as described previously [38] and separately reared in single wells of 24-well plates (4430300N, Orange Scientific) until they reached adulthood and were ready for wing dissection. For the fertility assays and the proof-of-principle spanning four generations, single pairs were crossed in separate glass tubes (LC84.1, Carl Roth).

### Wing dissection procedure

Individual adults from the Prl strain were transferred from the 24-well plate to the lid of a glass dish that was filled with ice and covered in parafilm. Upon paralysis due to the low temperature, the adult beetles were turned on the side and held in position with forceps. The tip of a micro prober was carefully slid sideward below the distal, slightly bent region of the right elytron to lift it from the abdomen and expose the right wing. Using the forceps, the wing was dragged forth laterally, pressed against the parafilm and cut as proximal as possible with a micro scalpel. If necessary, the left wing was dissected similarly. Subsequently, the adults were transferred to a non-cooled glass dish to recover from paralysis and then returned to their rearing well inside the 24-well plate.

### Survival and fertility assays

After eclosure, adults were given two to three weeks to mature. Either one or both wings were dissected from ten individuals (mixed sexes) in five replicates and survival was assayed one week after dissection. The sham control animals were also paralyzed, and the right elytron was also lifted from the abdomen, but the wing was not dissected. All surviving adults, dissected and sham, were provided with a partner of the opposite sex. After two additional weeks, progeny production was checked. A two-sample / two-tailed Student’s t-test was performed to determine significance, normal distribution was confirmed by the Shapiro-Wilk normality test [39].

### DNA extraction

#### Commercial kit

Genomic DNA from the whole body, one wing or both wings of individual adults was extracted by using the Blood & Tissue Kit (69504, Qiagen) according to the manufacturer’s instructions. Eluation volume was 15 μl. From the eluate, 1 μl for whole body and 5 μl for wings were used as a template for the PCR-based genotyping assay.

#### Custom method

Dissected wings were individually placed in 200 μl reaction tubes and stored on ice. After the dissection procedure, the wings were frozen at -80°C for 15 min and physically homogenized with a micro mortar. The fragments were covered with 10 μl of Proteinase K solution (500 μg/ml Proteinase K, 10 nM Tris-Cl, 1 mM EDTA, and 25 mM NaCl in double-distilled H_2_O), the mixture was incubated at 37°C for 1 h and then inactivated at 75°C for 20 min. From this solution, 1 μl was used as a template for the PCR-based genotyping assay.

### Molecular biology

#### Polymerase chain reaction (PCR) assays

All PCRs were performed with the Phusion polymerase (M0530L, New England BioLabs) in 20 μl reactions. Primer sequences are listed in S1 Table. PCR products were mixed with 4 μl 6× DNA gel loading dye (R0611, Thermo Fisher Scientific) and run on a 1% (wt/vol) agarose broad range (T846.3, Carl Roth) gel in TAE buffer at 100 V for 35 min. The GeneRuler DNA Ladder Mix (SM0331, Thermo Fisher Scientific) was used as the molecular weight size marker. A two-sample / two-tailed Student’s t-test was performed to determine significance, normal distribution was confirmed by the Shapiro-Wilk normality test [39].

#### Sequencing of PCR products

PCR products were purified using the NucleoSpin Gel and PCR Clean-up Kit (740609.250, Macherey-Nagel) according to the manufacturer’s instructions. Purification was followed by A-tailing using the Recombinant Taq DNA polymerase (10342020, Thermo Fisher Scientific), ligation into the pGEM-T Easy plasmid (A1360, Promega) and transformation into heat-shock competent DH5α *E*. *coli*. Plasmids were isolated with the NucleoSpin Plasmid Kit (740588.250, Macherey-Nagel) according to the manufacturer’s instructions. Control digestions were performed with NotI-HF (R3189L, New England BioLabs). The digested plasmids were run on a 0.8% (wt/vol) agarose broad range gel in TAE buffer at 100 V for 30 min. Plasmids that showed the correct pattern on the gel were sent for sequencing. Sequencing was performed initially with the T7 and SP6 primers, sequence-specific primers were used to continue sequencing when the initial results became available. The SB *white* allele sequence is provided as S1 Sequence, the Prl *white* allele sequence is provided as S2 Sequence.

## Results

### Wing dissection procedure, survival and fertility

**Fig 1:**
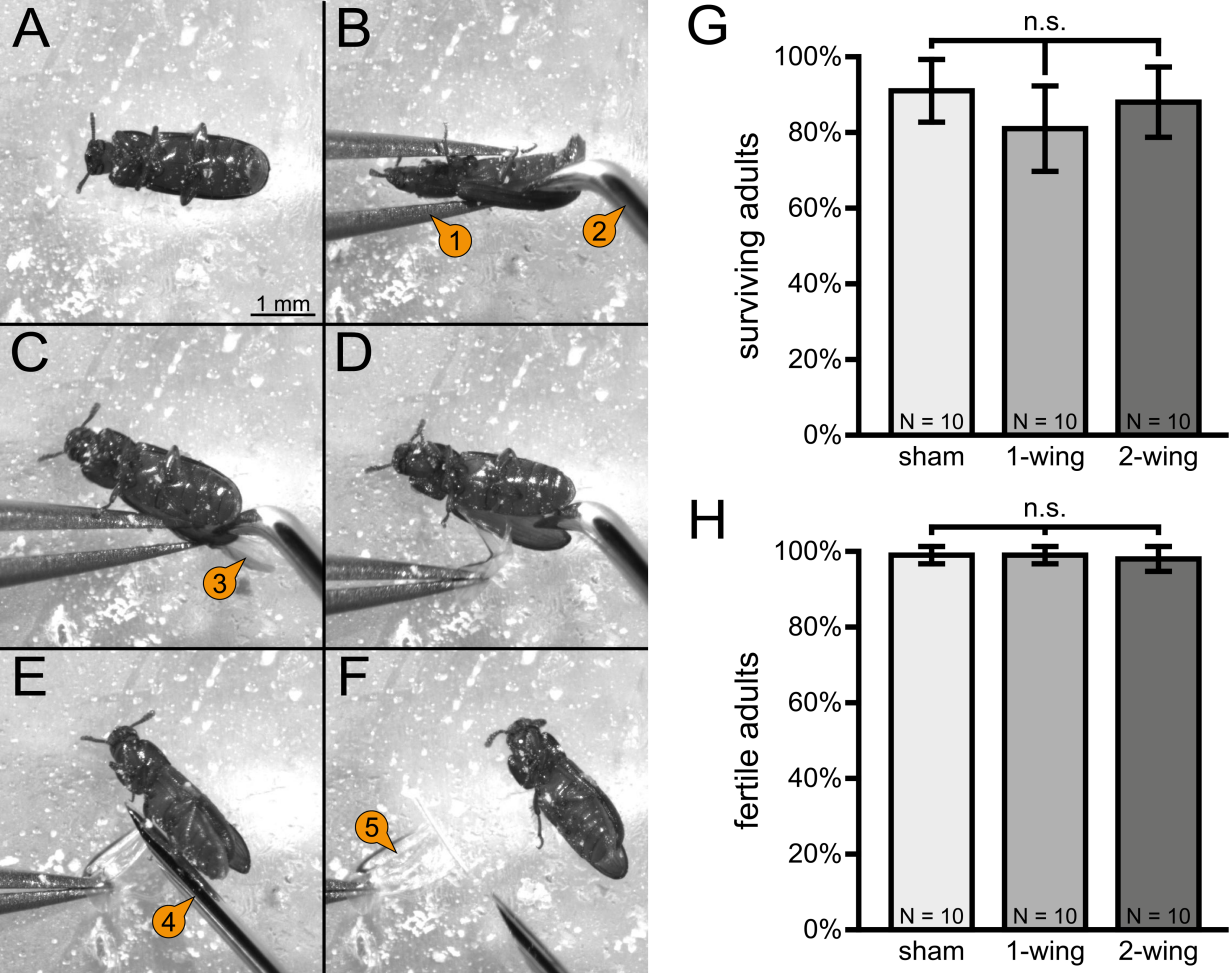
Wing dissection procedure and impact on survivability and fertility. **(A)** Adults are paralyzed by incubation on a parafilm-covered lid of a glass dish filled with ice for a few minutes. **(B)** The adult is held in position with a forceps (1) and a micro prober (2) is used to push the right elytron aside. **(C)** The micro prober is carefully inserted at the posterior region into the groove between the right elytron and the abdomen to expose the right wing (3). **(D)** The right wing is pressed onto the parafilm with the forceps. **(E)** Using a micro scalpel (4), the wing is cut as proximal as possible. **(F)** The dissected wing (5) is transferred to a 200 μl reaction tube and stored on ice. **(G)** Quantification of surviving adults from ten replicates with ten adults after the dissection of one or both wings. The sham control animals were also paralyzed, and their elytra were also lifted from the abdomen, but the wing was not dissected. No significant difference was found. Error bars represent standard deviation. **(H)** Quantification of fertile adults from ten replicates with the respective number of surviving adults after dissection of one or both wings. No significant difference was found. Error bars represent standard deviation.

In contrast to *D*. *melanogaster* and *A*. *mellifera*, the wings of *T*. *castaneum* are covered by protective elytra and thus not freely accessible. Therefore, we developed a ‘gentle’ procedure to dissect one or both wings from an adult beetle (Fig 1A–1F), which is comprehensively described within the Materials and Methods section. Removal of one or both wings has no significant effect on the survival rate (Fig 1G) and nearly all survivors were fertile after wing dissection (Fig 1H). To ensure that the presented ratios cannot be credited to a high personal skill level of a single experimenter, we compared the survival rate of adult beetles when either one or both wings were dissected by two independent experimenters. No significant difference in the performance was detected (S1 Fig).

### Proof-of-principle on the molecular level

**Fig 2:**
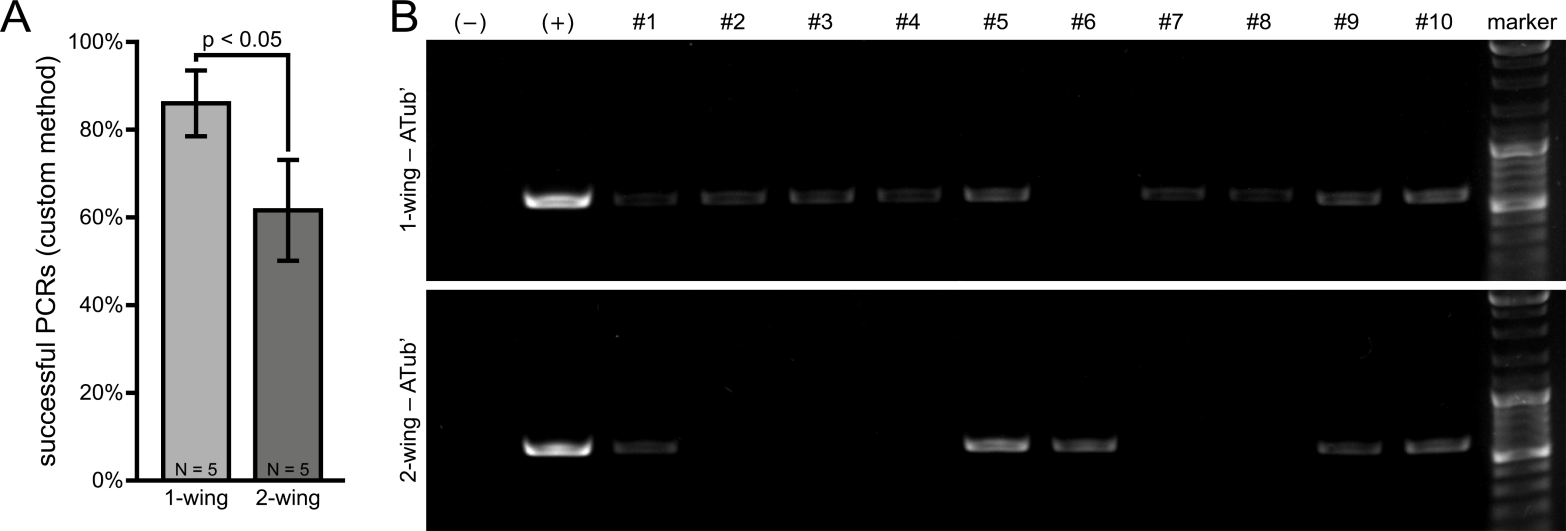
Successful PCRs on genomic DNA extracted from one or both wings by using the custom method. **(A)** Quantification of successful PCRs on the *alpha-tubulin 1* upstream regulatory sequence from five replicates with ten adults each using genomic DNA extracted from one or both wings as template. Significantly more successful PCRs could be performed when only one wing was digested. Error bars represent standard deviation. **(B)** Exemplary agarose gel electrophoresis images showing the PCR-based amplification of the *alpha-tubulin 1* upstream regulatory sequence (ATub’) for ten individual adults using DNA extracted from either one or both wings. The estimated PCR product size is 616 bp. The bright DNA marker bands (top to bottom) represent 3,000 / 1,000 / 500 bp. In the negative control (–), double-distilled H_2_O was used as template and in the positive control (+), DNA extracted from the whole body was used as template.

First, we assayed whether the amount of template obtainable from one or both wings by using a commercial DNA kit is sufficient for PCR-based genotyping. The primers were designed to target a part of the *alpha-tubulin 1* upstream regulatory sequence [40], which should result in a product with 616 bp length. However, the amount of tissue is about three orders of magnitude less than the recommended starting material and thus, the ratio of successful PCRs was inconveniently low (S2 Fig). Therefore, we developed a custom method for DNA extraction where either one or both wings were frozen, manually homogenized and digested in the same volume of Proteinase K solution. This approach was more effective in general, but with 86% to 62%, we obtained significantly more successful PCRs when only one wing was used (Fig 2).

### Sequencing of the San Bernadino and Pearl *white* alleles

**Fig 3:**
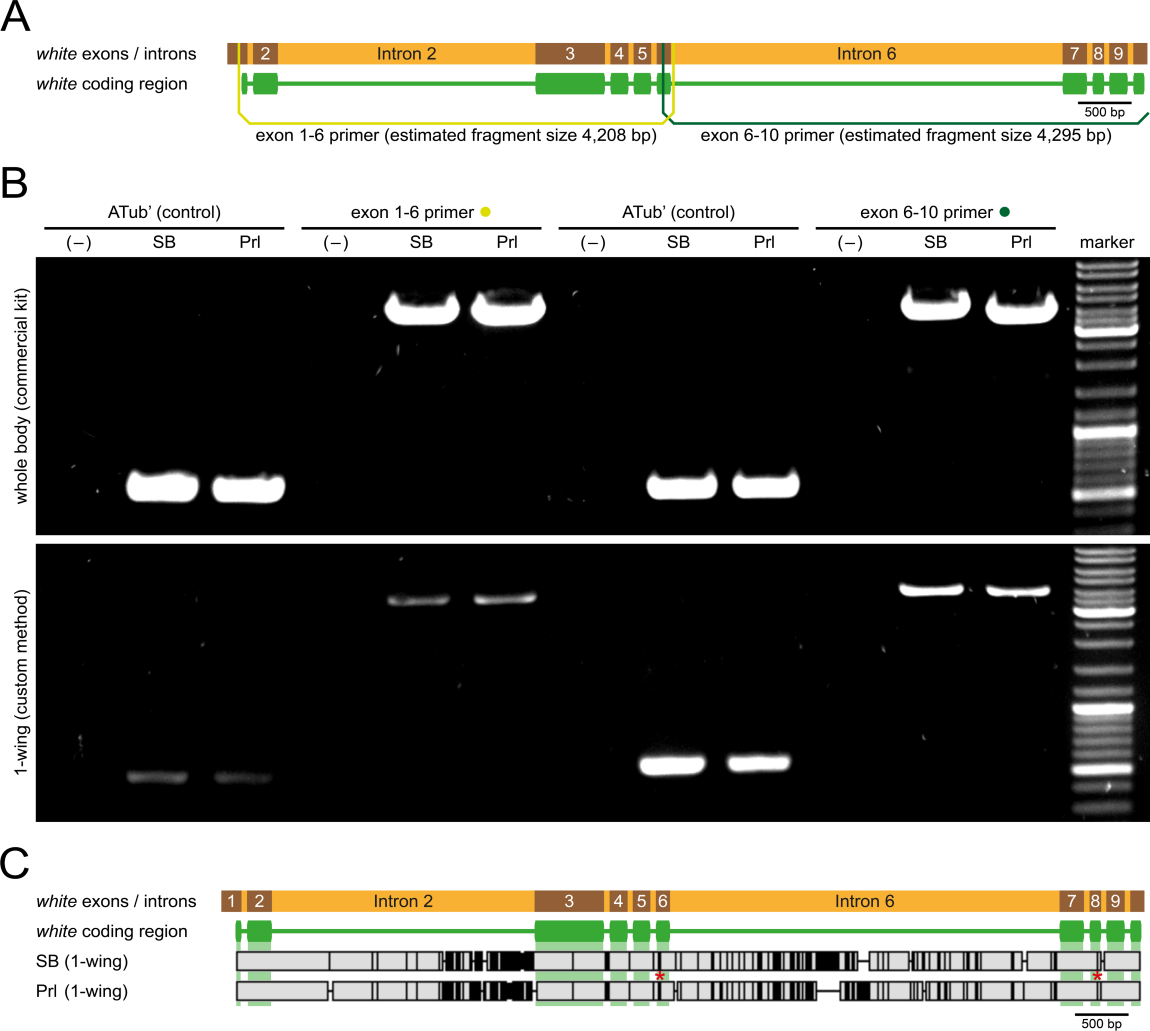
Sequencing of the SB and Prl *white* allele. **(A)** Exon / intron structure of the *white* gene (8,376 bp) with respective primer groups for extraction PCRs. **(B)** Agarose gel electrophoresis image showing the results for the extraction PCRs. Estimated PCR product size is 616 bp for the *alpha-tubulin 1* upstream regulatory sequence control (ATub’), 4,208 bp for the exon 1–6 primer group and 4,295 bp for the exon 6–10 primer group. Bright DNA marker bands (top to bottom) represent 3,000 / 1,000 / 500 bp. In the negative control (–), double-distilled H_2_O was used as template. **(C)** Alignment of the SB and Prl *white* sequences. Vertical black bars represent sequence deviations, horizontal black lines show insertions / deletions. The red asterisks mark the two mutations that lead to amino acid exchanges (Y437V and G573S).

Next, we tested whether DNA extracted with the custom method suffices to obtain the nucleotide sequence. We used the black-eyed SB wild-type strain and the white-eyed Prl recessive mutant strain. The current assumption is that the Prl phenotype is caused by a dysfunction of either *white* or *scarlet*, a pair of ABC transmembrane transporter genes that are co-localized in a tail-to-head fashion on chromosome LG9 [41,42]. According to the genomic sequence [13], which derives from the black-eyed GA-2 strain, the *white* gene contains ten exons distributed across a distance of 8,376 bp. We designed primers to amplify the whole coding sequence within two PCRs that cover 4,208 (exon 1–6 primer) and 4,295 bp (exon 6–10 primer), respectively (Fig 3A) and successfully amplified the target sequences from genomic DNA extracted from the whole body and from one wing for both, the SB and Prl strain (Fig 3B). The PCR products obtained for SB and Prl from wing tissue were subsequently sequenced, revealing nearly complete identity through the *white* coding sequence except for several silent mutations and two mutations that lead to an amino acid exchange, Y437V and G573S. However, a high number of insertions, deletions and substitutions were found within introns 2 and 6 (Fig 3C). We also compared the sequencing results from genomic DNA extracted from either the whole body or from one wing, which were nearly identical except for two single nucleotide polymorphisms in both, the SB (Panel A in S3 Fig) and Prl (Panel B in S3 Fig) strain. Additionally, we compared the SB (Panel A in S4 Fig) and Prl (Panel B in S4 Fig) allele sequences to the GA-2 reference [13]. For SB, three amino acid exchanges and comprehensive deviations in intron 6 were found, while for Prl, four amino acid exchanges (three in a similar position as for SB) and comprehensive deviations in introns 2 and 6 were found.

### Proof-of-principle spanning four generations

**Fig 4:**
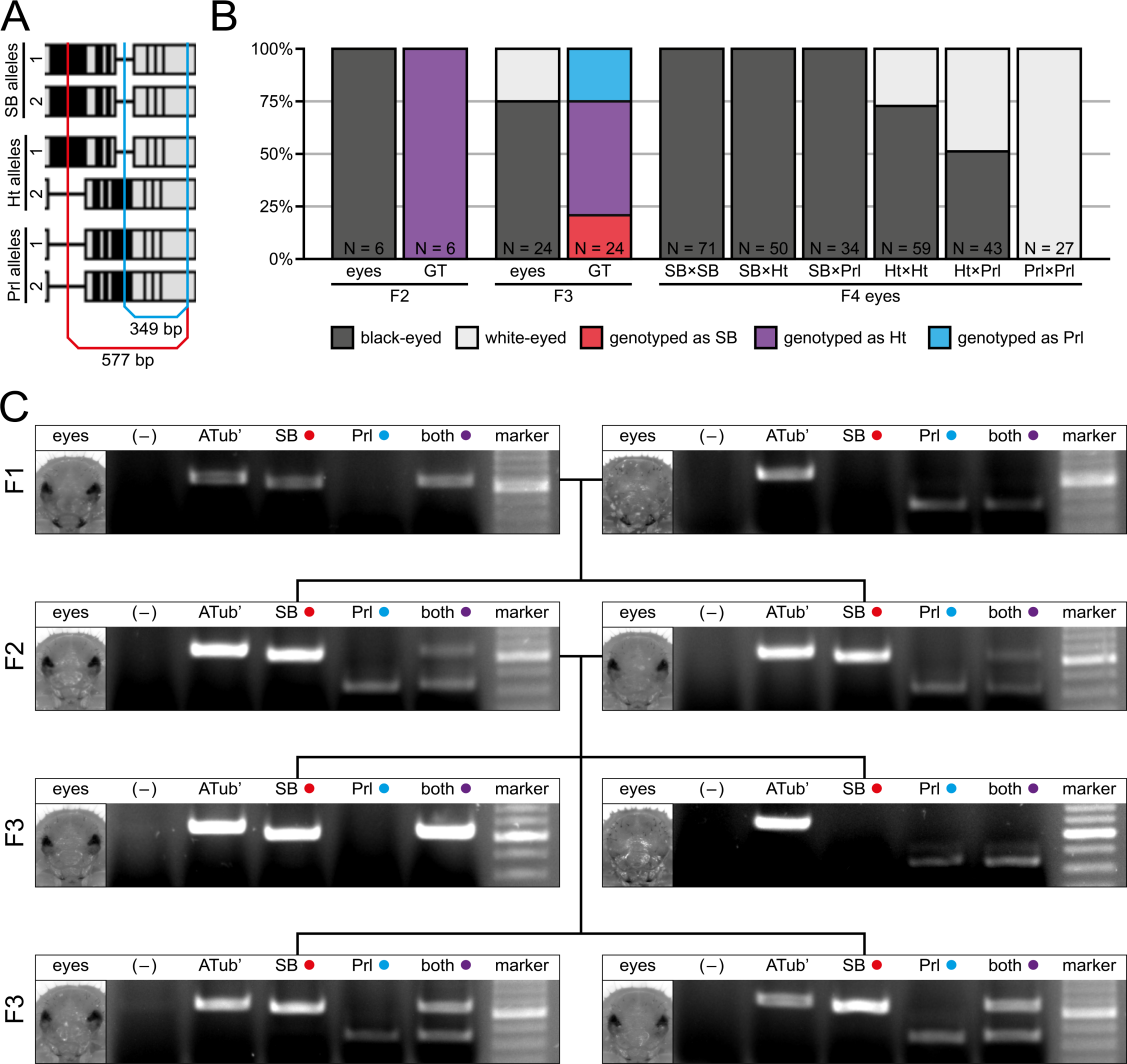
Proof-of-principle spanning four generations. **(A)** Primer group design for genotyping of either the SB (red lines) or Prl (blue lines) allele. **(B)** Eye pigmentation from G2 to G4 and genotyping results from G2 to G3. In the G1, a SB female was crosses with a Prl male, and the progeny was assayed phenotypically and their genotype was determined. **(C)** Exemplary results from the proof-of-principle assay from the G1 to G3. Estimated PCR product size is 616 bp for the *alpha-tubulin* 1 control (ATub’), 577 bp for the SB primer group, 349 bp for the Prl primer group and 577 as well as 349 bp for the primer group that contains both forward primers. Bright DNA marker band represents 500 bp. The negative control (–) used double-distilled H_2_O as template. Ht, heterozygote.

Our sequencing results of the *white* alleles do not properly reveal if the Prl allele is functional or if the white-eyed phenotype results from a lesion close to the C-terminal end in the co-localized *scarlet* gene as proposed by Grubbs *et al*. [42]. However, the obtained sequence data is adequate to design primer groups that allow the genotyping of the *white* / *scarlet* gene pair alleles for the SB and Prl strains based on the *white* intron 6 insertion / deletion pattern. The primer groups share the same reverse primer that attaches in an identical region, while the forward primers are different. The SB forward primer attaches in a region that is not present in the Prl allele and results in a 577 bp PCR product in the SB allele (Fig 4A, red lines). The Prl forward primer attaches in a region that is not present in the SB allele and result in a 349 bp PCR product on the Prl allele (Fig 4A, blue lines). We crossed a F1 SB female with a F1 Prl male and analyzed the F2 progeny phenotypically and via non-lethal genotyping using DNA extracted from one wing as a template. All F2 individuals showed black eyes and all were identified as SB/Prl heterozygotes (Fig 4B, first block). We crossed two F2 SB/Prl heterozygotes, which resulted in 75% progeny with black eyes. Out of those individuals, 20.8% were genotyped as SB, while 54.2% were genotyped as SB/Prl heterozygotes. The remaining 25% had white eyes and were genotyped as Prl (Fig 4B, second block). With the GF individuals, we performed six different control crosses to confirm the genotyping results of the F3 generation by the F4 eye phenotype (Fig 4B, third block). All experimentally obtained ratios agree with the theoretical Mendelian values, exemplary agarose gel electrophoresis images outlining the genotyping results for F1, F2 and F3 are shown in Fig 4C.

## Discussion

**Fig 5:**
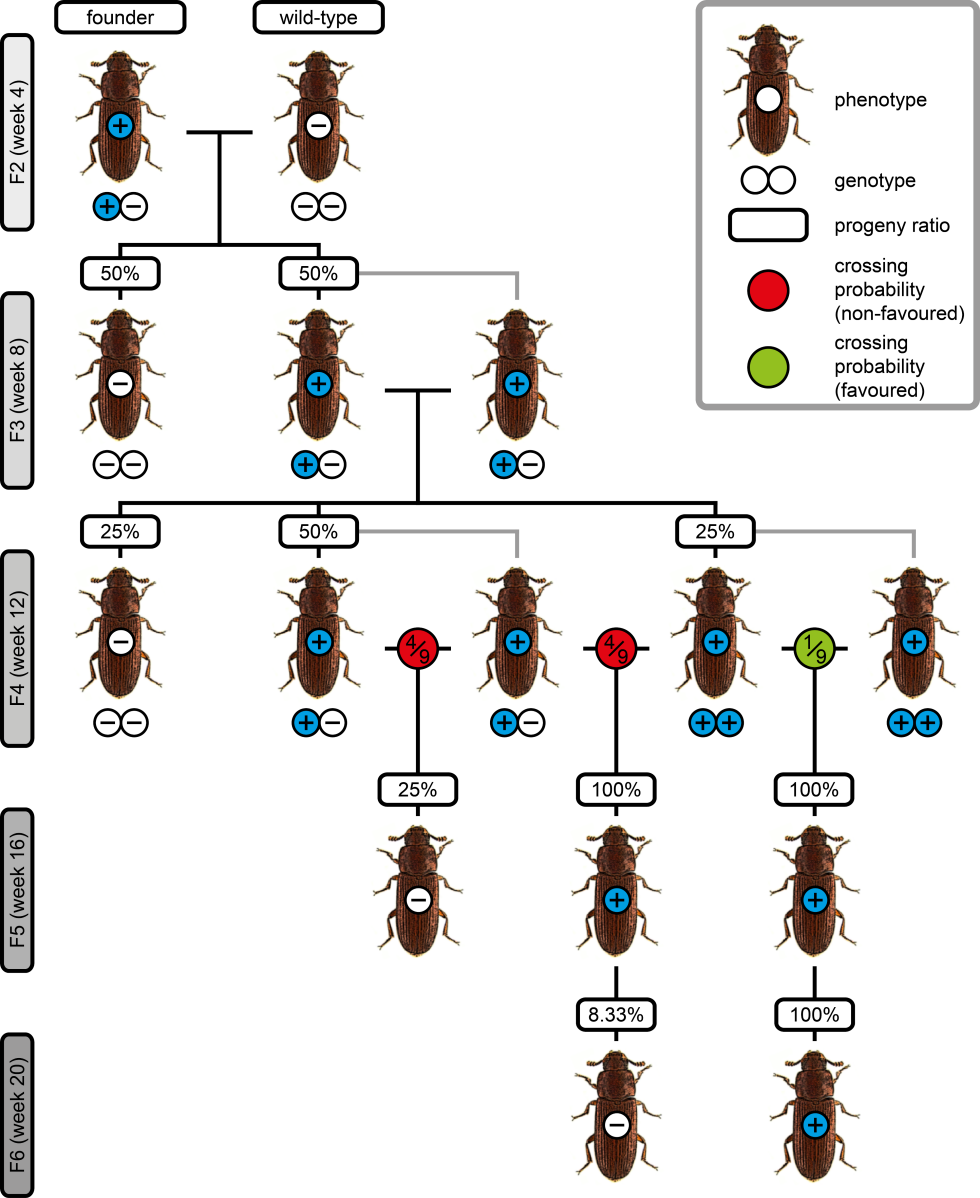
F2 to F5 crossing procedure for the establishment of homozygous cultures when the respective allele induces a dominant phenotype. F1 (not shown) is either the generation that was irradiated or treated with chemicals (mutants), the generation that was injected and thus has a mosaic-like integration pattern (custom-made transgenic lines) or the donor/helper hybrid generation (insertional mutagenesis screening). To establish a homozygous culture, a heterozygous F2 founder, which shows the respective phenotype, is outcrossed against the wild-type. In the F3, two heterozygous animals are crossed, which results in 25% wild-types, 50% heterozygotes and 25% homozygotes in the F4. Since heterozygotes and homozygotes are usually not distinguishable by the phenotype, individuals are crossed in single pairs without *a priori* knowledge of their allelic configuration and test crossings are performed to determine the genotypes. The genotypes of a F4 heterozygous/heterozygous pair can be identified by the phenotype distribution in the F5, since around 25% wild-types are expected. However, the genotypes of F4 heterozygous/homozygous and homozygous/homozygous pairs cannot be determined by inspecting the F5 since every descendant shows the phenotype. An additional F5 *en masse* cross is necessary to determine the genotypes of the F4 pair–no wild-types among the F6 progeny indicate that both F4 individuals were homozygous. This is a rather cumbersome process since statistically, only one out of nine F4 pairs consists of two homozygotes, and only four of those nine pairs can be discarded by analyzing the F5. Non-lethal genotyping allows to pair only homozygous animals in the F4, thus saving consumables (mainly rearing supplies) and manpower (to set up, cultivate and screen the F5 and F6 generations). Additionally, the process saves time, since awaiting the F5 and F6 is not necessary. At a rearing temperature of 30°C, the generation time is approximately 4 weeks, thus with non-lethal genotyping, homozygous cultures are available about 8 weeks more early.

In this study, we describe a viable and efficient wing dissection procedure for *T*. *castaneum* that more than eight out of ten beetles survive. The amount of DNA extracted from one wing performs reliably in PCRs and is sufficient for ten reactions. However, doubling the amount of tissue, *i*.*e*. digesting two wings in the same volume of Proteinase K solution, decreases the efficiency. We assume that although the amount of DNA template is twice as high, the increase in organic debris as a by-product of the digestion reaction interferes with the subsequent PCR. Even for distances of around 4,000 bp, the amount of PCR product is adequate for sequencing. Thus, our method facilitates all experimental workflows, in which the exact genetic configuration of individual living beetles has to be known. We assume that non-lethal genotyping of *T*. *castaneum* will be of use in the following scenarios:

- The most trivial solution for stock keeping of non-lethal alleles is the establishment of homozygous cultures. For alleles that induce a dominant phenotype, *e*.*g*. certain mutants [43], custom-made transgenic lines for applications such as fluorescence live imaging [44], or enhancer trap lines that are result from large-scale assays such as insertional mutagenesis screenings [33], the establishment procedure from a single founder is rather complex. If relevant progeny genotypes have to be identified via test crossing, four generations are necessary to distinguish homozygous from mixed cultures. In contrast, our method allows the identification of homozygous animals after two generations (Fig 5).
- Stock keeping of cultures that carry embryonic recessive lethal alleles is laborious since heterozygous animals are usually phenotypically inconspicuous and appropriate balancers are not always available [4,45]. Respective technical protocols [46] suggest test crossing followed by phenotypical identification of mutant phenotypes within the eggs. With our non-lethal genotyping approach, both steps can be replaced by determining the genotype via molecular biology-based assays.
- Genome engineering with the CRISPR/Cas9 nuclease system has been established for *T*. *castaneum* [30]. However, respective assays require the screening of a certain number of potential founders to identify appropriately altered alleles. Typically, founders have to be crossed against wild-types and sequence analysis has to be performed with the progeny. With our technique, the sequences of potentially altered alleles can be determined directly in the founders and only the favorable individuals are subsequently crossed against the wild-type to secure the allele.
- Besides developmental biology, genetics and agricultural-related research, *T*. *castaneum* is also used in behavior experiments [47–49]. The proposed method also improves workflows where genotype and behavior need to be correlated–the genotypes can be determined before the respective individuals are used in assays.

Protocols for non-lethal genotyping by extracting genomic DNA from dissected legs has been shown for *D*. *melanogaster* [31] and two wasp species [50]. In *T*. *castaneum*, we consider the wing as the ideal tissue choice for multiple reasons: Firstly, under laboratory culture conditions, adult beetles scarcely fly but primarily scuttle and/or dig tunnels into the flour. Secondly, the dissection wound is properly covered and not exposed to pollutants or pathogens. Thirdly, legs are an essential factor for the mating behavior of *T*. *castaneum* since males rub their legs against the elytra of the partner during copulation. The oviposition rate of females that were paired with males, which hat their tarsi ablated was significantly decreased [51–53]. This is in direct conflict with the primary motivation for our method, *i*.*e*. genotyped individuals with the desired allele(s) have to produce sufficient progeny.

For *D*. *melanogaster*, quantitative data on the performance of non-lethal genotyping is not available [31], but our ratio of successful PCRs, which is about 86%, is comparable to the 87.5% that have been obtained for *A*. *mellifera* workers when a similar genomic DNA extraction procedure was used [32]. Due to the protective elytra, the extraction of wing tissue from *T*. *castaneum* is experimentally slightly more challenging. With some training, the wing dissection time per individual reduces to two to three minutes. Including digestion, PCR and electrophoresis, the complete process for up to fifty adults is completed in less than an afternoon.

The nucleotide sequences of the *white* alleles from the SB wild-type and Prl mutant strains do not provide a clear evidence for the functionality of the respective genes. The coding sequence in both alleles appears intact and severe deviations are only found in the intron sequences. These finding are consistent with the conclusions from Grubbs *et al*. [42], which proposed that not the *white*, but the *scarlet* gene is dysfunctional in the Prl strain. To completely verify this assumption, a complementation assay needs to be performed where functional variants of both, the *white* and the *scarlet* gene, are expressed in the Prl strain via transgenesis.

Non-lethal genotyping is a valuable addition to the methodological arsenal for *T*. *castaneum* and an adaptation of the proposed procedure for other coleopteran species should be convenient. Compared to the known alternatives, our approach has a high potential to save manpower, consumables and time. However, our approach is invasive, which might interfere with certain experimental strategies. In such scenarios, it may be necessary to establish non-invasive genotyping protocols, for example by extracting genomic DNA from exuviae as shown for *A*. *mellifera* [54]. Exuviae mainly consist of extra-cellular proteins and polysaccharides, but usually, a few ectodermal cells from the trachea system and the digestive tract detach during the molting process.

## Supporting information

S1 Fig Comparison of survival rate when either one or both wings were dissected by two experimenters. **(A)** Quantification of surviving adults from five replicates with ten adults after the dissection of one wing. The sham control animals were also paralyzed, and their elytra were also lifted from the abdomen, but the wing was not dissected. No significant difference was found. Error bars represent standard deviation. **(B)** Quantification of surviving adults from five replicates with ten adults after the dissection of both wings. The sham control animals were also paralyzed, and their elytra were also lifted from the abdomen, but the wing was not dissected. No significant difference was found. Error bars represent standard deviation. Exp, experimenter.
(TIF)

S2 Fig Successful PCRs on genomic DNA extracted from one or both wings by using a commercial DNA extraction kit. **(A)** Quantification of successful PCRs on the *alpha-tubulin 1* upstream regulatory sequence from five replicates with ten adults each using genomic DNA extracted from one or both wings as template. No significant difference was found. Error bars represent standard deviation. **(B)** Exemplary agarose gel electrophoresis images showing the PCR-based amplification of the *alpha-tubulin 1* upstream regulatory sequence (ATub’) for ten individual adults using DNA extracted from either one or both wings. The estimated PCR product size is 616 bp. The bright DNA marker bands (top to bottom) represent 3,000 / 1,000 / 500 bp. In the negative control (–), double-distilled H_2_O was used as template and in the positive control (+), DNA extracted from the whole body was used as template.
(TIF)

S3 Fig Comparison of sequencing results from genomic DNA extracted either from the whole body or from one wing for SB and Prl. **(A)** Comparison for the SB strain. Vertical black bars represent sequence deviations. Two deviations were found within intron 2. **(B)** Comparison for the Prl strain. Vertical black bars represent sequence deviations. Two deviations were found, one within intron 6, and one in exon 8. The deviation in exon 8 accounts for the G573S amino acid exchange (S4 Fig).
(TIF)

S4 Fig Comparison of the SB and Prl *white* gene sequences to the GA-2 standard. **(A)** Comparison for the SB allele. Vertical black bars represent sequence deviations, horizontal black lines show insertions / deletions. Compared to GA-2, the SB *white* allele has multiple insertions, deletions and substitutions mainly within intron 6. The deviations within the coding sequence are primarily silent, except for three amino acid exchanges, T34S, L80F and D437Y, as marked by the red asterisks. **(B)** Comparison for the Prl allele. Vertical black bars represent sequence deviations, horizontal black lines show insertions / deletions. Compared to GA-2, the Prl *white* allele has multiple insertions, deletions and substitutions mainly within introns 2 and 6. The deviations within the coding sequence are primarily silent, except for four amino acid exchanges T34S (similar to SB), L80F (similar to SB), D437V (same position as in the SB sequence, but results in a different amino acid) and G573S (not present for SB), as marked by the red asterisks.
(TIF)

S1 Table Primer group list. ATub’, *alpha tubulin 1* upstream regulatory sequence; ExPCR, primer for extraction PCRs; FD, forward; RV, reverse.
(XLSX)

S1 Sequence SB *white* allele (genomic DNA extracted from one wing; 8,506 bp). (TXT)

S2 Sequence Prl *white* allele (genomic DNA extracted from one wing; 8,372 bp). (TXT)
