# Supplementary figures and images for: Non-lethal genotyping of Tribolium castaneum adults using genomic DNA extracted from wing tissue
Source: PLoS One. 2017 Aug 11;12(8):e0182564. doi: 10.1371/journal.pone.0182564 (PMC5553768; doi:10.1371/journal.pone.0182564)

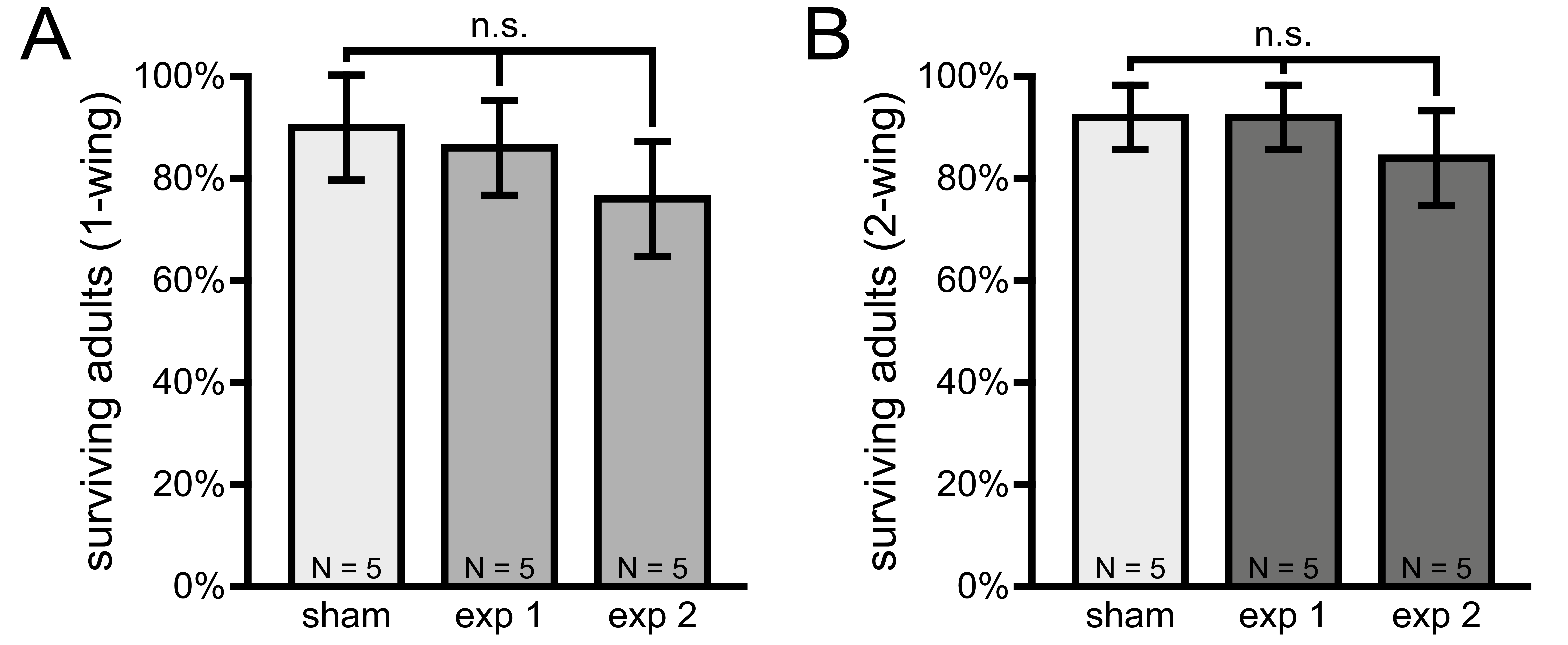

Supplement: S1 Fig — (A) Quantification of surviving adults from five replicates with ten adults after the dissection of one wing. The sham control animals were also paralyzed, and their elytra were also lifted from the abdomen, but the wing was not dissected. No significant difference was found. Error bars represent standard deviation. (B) Quantification of surviving adults from five replicates with ten adults after the dissection of both wings. The sham control animals were also paralyzed, and their elytra were also lifted from the abdomen, but the wing was not dissected. No significant difference was found. Error bars represent standard deviation. Exp, experimenter. (TIF) [file pone.0182564.s001.tif]

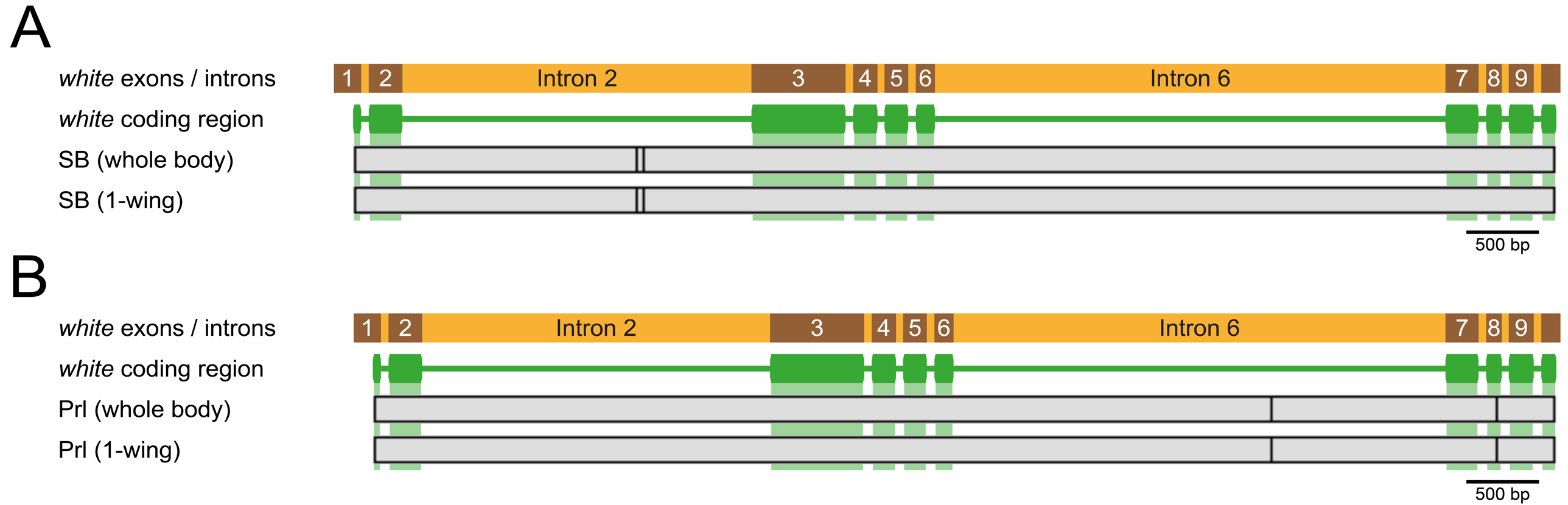

Supplement: S3 Fig — (A) Comparison for the SB strain. Vertical black bars represent sequence deviations. Two deviations were found within intron 2. (B) Comparison for the Prl strain. Vertical black bars represent sequence deviations. Two deviations were found, one within intron 6, and one in exon 8. The deviation in exon 8 accounts for the G573S amino acid exchange (S4 Fig). (TIF) [file pone.0182564.s003.tif]

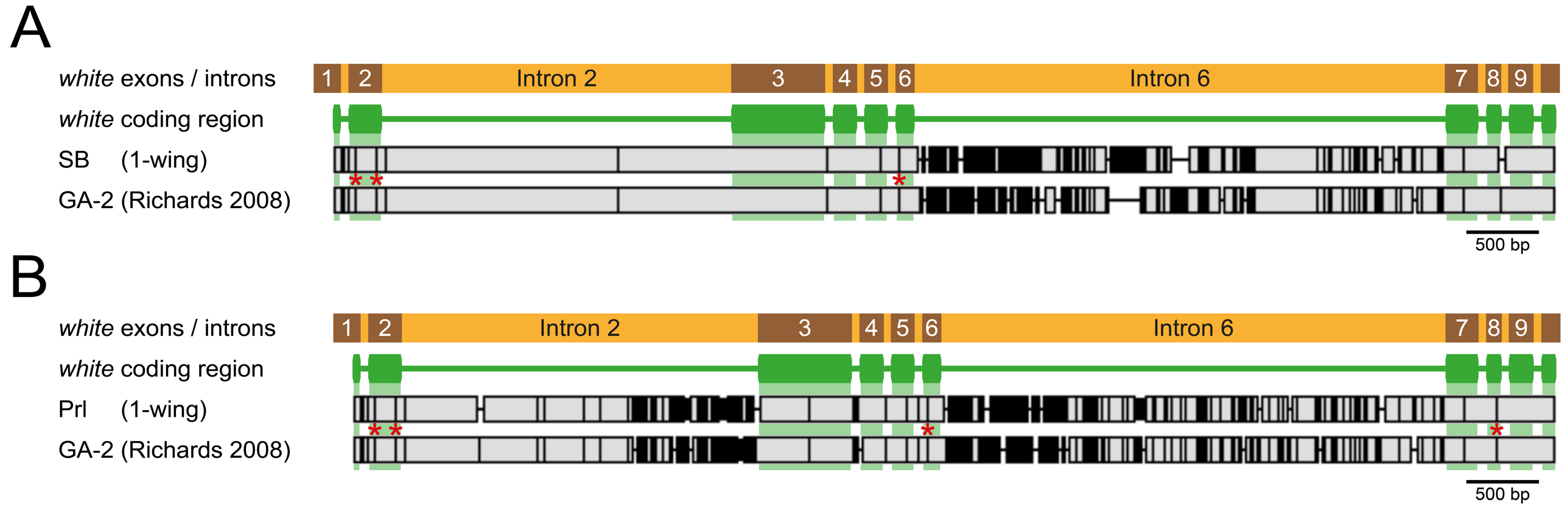

Supplement: S4 Fig — (A) Comparison for the SB allele. Vertical black bars represent sequence deviations, horizontal black lines show insertions / deletions. Compared to GA-2, the SB white allele has multiple insertions, deletions and substitutions mainly within intron 6. The deviations within the coding sequence are primarily silent, except for three amino acid exchanges, T34S, L80F and D437Y, as marked by the red asterisks. (B) Comparison for the Prl allele. Vertical black bars represent sequence deviations, horizontal black lines show insertions / deletions. Compared to GA-2, the Prl white allele has multiple insertions, deletions and substitutions mainly within introns 2 and 6. The deviations within the coding sequence are primarily silent, except for four amino acid exchanges T34S (similar to SB), L80F (similar to SB), D437V (same position as in the SB sequence, but results in a different amino acid) and G573S (not present for SB), as marked by the red asterisks. (TIF) [file pone.0182564.s004.tif]
